# Has Drug Repurposing Fulfilled Its Promise in Acute Myeloid Leukaemia?

**DOI:** 10.3390/jcm9061892

**Published:** 2020-06-17

**Authors:** Debora Valli, Alicja M. Gruszka, Myriam Alcalay

**Affiliations:** 1 Department of Experimental Oncology, Istituto Europeo di Oncologia IRCCS, Via Adamello 16, 20 139 Milan, Italy; debora.valli@ieo.it (D.V.); myriam.alcalay@ieo.it (M.A.); 2 Department of Oncology and Hemato-Oncology, University of Milan, Via Festa del Perdono 7, 20 122 Milan, Italy

**Keywords:** acute myeloid leukaemia, drug repurposing, candidate compounds

## Abstract

Drug repurposing is a method of drug discovery that consists of finding a new therapeutic context for an old drug. Compound identification arises from screening of large libraries of active compounds, through interrogating databases of cell line gene expression response upon treatment or by merging several types of information concerning disease–drug relationships. Although, there is a general consensus on the potential and advantages of this drug discovery modality, at the practical level to-date no non-anti-cancer repurposed compounds have been introduced into standard acute myeloid leukaemia (AML) management, albeit that preclinical validation yielded several candidates. The review presents the state-of-the-art drug repurposing approach in AML and poses the question of what has to be done in order to take a full advantage of it, both at the stage of screening design and later when progressing from the preclinical to the clinical phases of drug development. We argue that improvements are needed to model and read-out systems as well as to screening technologies, but also to more funding and trust in drug repurposing strategies.

## 1. Introduction: What Is Drug Repurposing?

Drug repurposing, or its variant-drug repositioning, is an attractive drug discovery approach whereby the exploitation of an already-approved or investigational drug for a new therapeutic indication is attempted [1,2,3,4]. Synonyms include reprofiling and re-tasking, whilst some authors use the terms “repurposing” and “repositioning” interchangeably. Since 2011, a large number of drug repurposing screens have been recorded, as attested to by the number of published papers, making drug repurposing a core strategy in drug development.

To date, most of the successfully repurposed drugs were identified serendipitously through the analysis of their side effects. However, drug repurposing requires systematic, high-throughput and rational methods [5]. Novel methods to recognize potential drugs to be repurposed are being developed and are indispensable (examples reviewed in [6,7]). Both experimental and computational repurposing approaches can be used. Experimental methods are comprised of two types of screens: target-based screens and phenotypic screens. The former identify novel targets of old drugs through binding assays to detect target interactions, mass spectrometry or affinity chromatography; the latter assess cell behaviour (e.g., growth/death) exploiting in vitro or in vivo phenotypic screening [8]. To date, at least ten compound collections are available for screening, including investigational agents, Food and Drug Administration (FDA)-approved drugs or compounds in advanced-phase clinical trials [7]. Computational methods are based on analyses of big data (e.g., gene expression, chemical structure, proteomic and genomic data). Each drug has a specific transcriptomic signature, chemical structure and adverse effect profile. Similarities in transcriptomic signatures may indicate that drugs share therapeutic applications regardless of their chemical structure [9,10]. Analogously, drugs that have similar chemical structure could share biological activity [11]. Moreover, similar adverse effects caused by different drugs may suggest that they act on the same targets or pathways [12,13]. Molecular docking strategy identifies binding site complementarity between a drug and a target [14]. Taking advantage of genome-wide studies and network analyses, genes/proteins can be inferred as targets in a specific disease [15]. Retrospective clinical analysis is also a powerful tool to find repurposing opportunities by interrogating data from sources such as electronic health records, post-marketing surveillance data and clinical trials results [16].

The most important advantage of repurposing is the reduction of time and costs necessary to deliver compounds to patient’s bedside. The development of a new drug can take 10–17 years at an estimated cost of 2 or 3 billion dollars from research to marketing approval. The reported timeframe for repurposing, instead, equals approximately six years with a clear positive impact on costs [17]. Together with shortening processing time, repurposing also reduces the failure rate of drug development. New drugs often fail to achieve FDA approval because they lack efficacy or turn out to be unsafe for patients, whereas a drug being repurposed offers a high level of safety as its pharmacokinetics, toxicity, bioavailability, dosage, administration, off-target and adverse effects are already known [1,2]. De novo drug discovery is made up of multiple steps: efficacy, toxicity, pharmacokinetics and pharmacodynamics are tested by performing preclinical in vitro and in vivo studies. Next, agents are tested on patients, focusing on compounds’ safety (phase I clinical trials) and efficacy (phase II clinical trials). The final step (phase III clinical trials) consists of randomised studies that compare the efficiency of the new treatment versus standard treatment or dosage tuning [18]. Repurposing, instead, skips some of the preclinical and toxicity (phase I) testing. Phase II and III clinical trials are still necessary and represent the critical bottle-necks as they exclude 68% and 40% of compounds, respectively [17]. The concepts of saving time, avoiding phase I trials and working with safe compounds have become the three dogmas of drug repurposing.

There are hurdles and limitations specific to re-tasking projects. The drug to be repurposed often acts through a new mechanism of action, on new molecular targets or both [19], therefore specific assays to measure its activity are indispensable. Moreover, a drug can elicit the desired pharmacological effect at concentrations that are higher than those used in the “old” therapeutic setting or reach anti-cancer effects only in combination with existing chemotherapeutic/new repurposed agents. In such cases, a new evaluation of absorption, distribution, metabolism, excretion, toxicity and side effects, should be performed [20]. Other barriers to drug repurposing include patent exclusivity and intellectual property rights. Usually, drugs to be repurposed are at the end of their patent or are generic agents manufactured and sold by multiple companies at a low cost. An investment is necessary to extend the license of a generic drug to patent its new use. New therapeutic indications of the off-patent drug must be innovative, supported by reliable data, unpublished and not suggested in publications. A sponsor, who owns the patent for a compound, can invest in it hoping for financial gain, whilst a generic compound is much less interesting [21]. A generic manufacturer, who invests in a compound to be repurposed, may be disadvantaged compared to their competitors as the cost of the drug could increase. In addition, no financial incentives to carry out clinical trials of drugs to be repurposed exist; there is no pressure for official approval, no market authorization or inclusion in clinical practice resulting in damage to public health and cancer patients.

The review presents the state-of-the-art drug repurposing approach in acute myeloid leukaemia (AML), analyses development times and poses the question of how to exploit this approach, both at the stage of screening design and later when progressing from the preclinical to the clinical phases of drug discovery.

## 2. Drug Repurposing in AML

AML is a heterogeneous group of clonal haematopoietic malignancies underlain by a failure to differentiate and over-proliferation in the stem cell/progenitor compartment producing non-functional blasts [22]. AML’s heterogeneity can be explained by the diversity of genotypes encountered [23] and epigenetic changes. Prognosis and response to treatment varies amongst the diverse subtypes, but by-and-large continues to be unsatisfactory as the therapeutic landscape in AML has remained relatively unchanged over the years. The gold standard of treatment is the “7+3” regimen consisting of seven days of cytarabine and three of anthracycline. Recently, eight de novo drugs received FDA approval for AML treatment. They comprise midostaurin, gilteritinib, enasidenib, ivosidenib, venetoclax, daurismo, CPX-351 and gemtuzumab ozogamicin [24,25,26]. However, the persisting unmet clinical need to increase cure rates in AML using new efficient therapeutics and the unaffordable prices of traditionally-developed anti-cancer drugs spurred the exploration of repurposing strategies for AML treatment [27].

Drug repurposing can be divided into ”soft repurposing” (otherwise known as repositioning) and ”hard repurposing” [28]. The former is based on the re-use of an existing anti-cancer drug in a novel oncological setting, while the latter involves the use of non-cancer drugs to fight cancer. Although both avenues have been trod in AML, this review will dedicate more space to hard repurposing.

### 2.1. Drug Repositioning (or Soft Drug Repurposing)

Drug repositioning seems intuitive and meets less resistance than hard repurposing. Soft repurposing in AML concerns clofarabine, cladribine, actinomycin D, azacytidine, melphalan, hydroxyurea and arsenic trioxide.

Clofarabine, a purine nucleoside antimetabolite structurally related to cladribine, is a case of soft repurposing as it was initially approved by the FDA for the treatment of children with relapsed or refractory acute lymphoblastic leukaemia (ALL) [29]. In the new therapeutic setting, it is used in combination with cytarabine, idarubicin, or both [30]. Cladribine resulted in being an effective anti-AML therapying in a meta-analysis of 10 clinical trials [31]. It improves clinical outcomes with no adverse effects when added to standard therapy [32]. Recently, the treatment of a 60-year-old AML patient bearing *NPM1* mutation with actinomycin D, an antibiotic approved for the treatment of rare cancers, led to a complete remission (CR) lasting 14 months. Future studies are necessary to assess the efficiency of actinomycin D in different AML subtypes [33]. Azacytidine (or 5-azacytidine), a pyrimidine nucleoside analogue that impairs DNA methylation, previously used for the treatment of myelodysplastic syndromes, was repurposed and approved by the European Medicines Agency (EMA) as an anti-AML therapy for patients older than 64 years and with < 20% bone marrow blasts in 2008. The approval was expanded in 2015 to include patients with blast percentage greater than 30% [34]. Melphalan, an alkylating agent used for the treatment of multiple myeloma, retinoblastoma, melanoma and ovarian cancer, is still under investigation as a potential treatment against AML. In 2000, two cases of elderly AML patients achieved CR when treated, albeit relapsed showing therapy-related chromosomal abnormalities and become resistant to treatment [35]. Successful results were obtained since using low-doses of melphalan in elderly patients with relapsed/refractory AML. The patients achieved CR, partial remission (PR) or disease stabilisation and the overall survival (OS) was extended. Currently, melphalan is considered to be a safe and efficient therapeutic strategy with mild adverse effects, particularly, after hypomethylating agents and in the absence of a complex karyotype [36]. Hydroxyurea is an antimetabolite used to treat melanoma, chronic myelocytic leukaemia, ovarian and head and neck cancer; in AML, it was tested in combination with cytarabine. Hydroxyurea improved the outcome, especially, in samples with high levels of SAMHD1 enzyme without causing additional side effects. AML patients who express high levels of SAMHD1 are resistant to cytarabine treatment as it breaks the active metabolite of cytarabine, ara-CTP [37]. Arsenic trioxide is used for the treatment of acute promyelocytic leukaemia (APL) resulting in CR rate of 85% in relapsed patients. It seemed ineffective in non-APL AML patients both as monotherapy and in combination with standard treatment [38,39,40]. Preliminary results from a clinical trial to evaluate the effect of arsenic trioxide combined with ascorbic acid suggest that the combination has an anti-leukaemia activity in untreated-AML patients providing an alternative approach to treat AML patients who cannot undergo intensive chemotherapy [41]. Two recent studies suggested the potential usefulness in AML treatment of glucocorticoids, such as dexamethasone, otherwise used for the treatment of multiple myeloma [42,43]. Cytarabine-resistant cell lines and chemo-refractory AML patient samples without *FLT3* mutation seemed to be more sensitive to glucocorticoids than cytarabine-responsive cells [42]. Additionally, glucocorticoids displayed potent and selective activity against AML cells bearing t(8;21) translocation resulting in *RUNX1-ETO* fusion formation, and strong synergy when combined with chemotherapeutics [43]. Amongst the anti-infectious drugs used in hematopoietic stem cell transplantation protocols, salinomycin, a coccidiostat ionophore, is selectively active against AML and mixed lineage leukaemia-rearranged human cell lines, primary murine cells and patient samples.

Interestingly, salinomycin treatment of primary mouse leukaemia cells resulted in loss of leukaemia repopulation ability following transplantation [44]. Finally, Remsing Rix and colleagues demonstrated that tivantinib, an advanced clinical candidate, specifically inhibits GSK3α and to a lesser extent GSK3β in lung cancer cells [45]. The efficacy of tivantinib was thus tested in AML treatment as GSK3α is reported to be involved in AML development [46]. Tivantinib induced cell cycle arrest, apoptosis and differentiation of AML cell lines. Moreover, tivantinib synergised with ABT-199 BCL-2 inhibitor [47].

### 2.2. (Hard) Drug Repurposing

**Figure 1:**
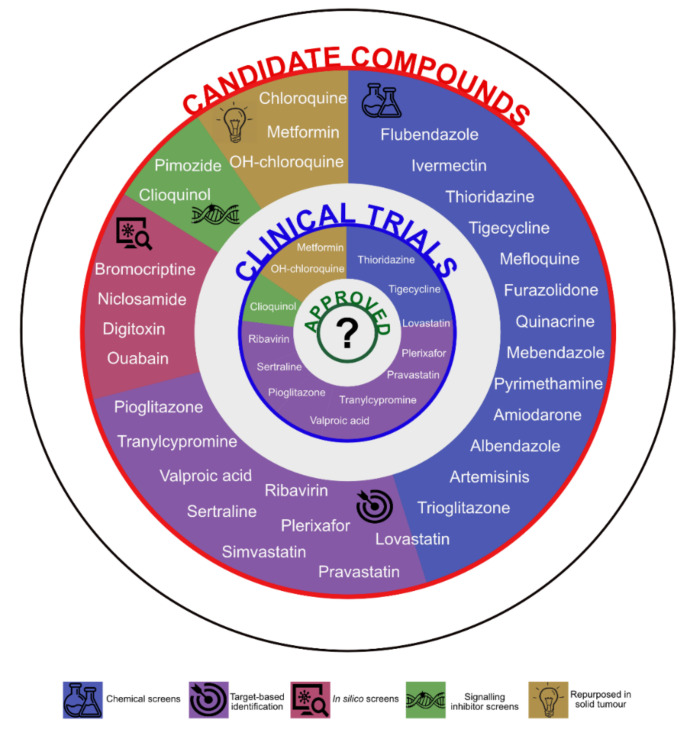
Candidate compounds to treat acute myeloid leukaemia (AML) obtained through a host of methods tested in preclinical (outer circle) and clinical setting (inner circle).

The chief example of hard repurposing in the field of haematology is thalidomide, a compound developed to alleviate morning sickness during pregnancy, withdrawn from the market due to its teratogenic properties and now approved for the treatment of multiple myeloma [48]. In AML, the identification of compounds to be repurposed occurred in three major ways: in the wake of a repurposing attempts in solid tumours, driven by the discovery of compounds being inhibitors of specific targets (i.e., receptors or enzymes deregulated in AML) or through unbiased screens of compound collections (Figure 1).

Metformin, chloroquine and hydroxychloroquine have been initially repurposed in solid tumours. Statins (simvastatin, pravastatin and lovastatin), valproic acid (VPA) anti-convulsion medicine, sertraline and tranylcypromine derivative antidepressants, pioglitazone metabolic regulator, ribavirin and AMD3100 antiviral agents were found to specifically inhibit molecular processes important for the growth of leukaemia. There is also epidemiological evidence of lower cancer incidence in patients under chronical medication in the case of some of the agents.

#### 2.2.1. Screening and Hits

In vitro and in silico screening projects pinpointed 19 compounds to be repurposed in AML. Nine large-scale and two small-scale chemical screens have been reported (Table 1). The large-scale efforts exploited the Prestwick, Library of Pharmacologically Active Compounds (LOPAC), Screen Well, National Institutes of Health (NIH) Clinical Collection, Canadian Compound Collection libraries or combination thereof, whilst others opted for small (up to 300 compounds) custom libraries of on- and off-patent drugs, mainly antimicrobials and metabolic regulators. Library agents combined with all-trans retinoic acid (ATRA) or ABT-737 BCL2 inhibitor were tested in two experimental settings. The readout in the vast majority of studies was that of cell viability at 24, 48 or 72 h with various cut-offs. The very first screening study used a model system to assess cyclin D2 transactivation status identifying its inhibitors through a luciferase assay and evaluating cell viability. STAT5 signalling inhibition or colony formation potential upon treatment were also investigated. The concentrations applied ranged from 1 to 50 μM; both single and multiple dose settings are reported. The model systems included common human AML cell lines (HL60, OCI-AML2, KG1a, U937), AML cell lines with leukaemic stem cell (LSC) features (TEX, M9-ENL1, AML 8227), murine bone marrow stem/progenitor cells transduced with oncogenes of interest (*AML1-ETO*, *MN1*, *MLL-AF9*, *HOX9A-Meis1*) or a leukaemic niche-like system. One study screened the LOPAC compounds against four primary AML samples and peripheral blood mononuclear cells from a healthy donor. Neoplastic and normal human pluripotent stem cells were used in a screen to identify differentiating agents within a custom library of 590 compounds.

Three groups approached drug repurposing in AML from the in silico angle taking advantage of the Connectivity Map (cmap; Broad Institute) or 2D structure similarity analysis; the most recent of the three paired a cmap (Built 2.0) query to select compounds that kill LSCs whilst sparing normal haematopoietic counterparts to balance between healthy and leukaemic stem cells with an in vitro secondary screen. Not all studies reported the number (or percentage) of hits identified and only three published their shortlists. Hits comprised 1.6 to 11.5% of agents present in the library analysed. Clearly, this datum depends on experimental system and cut-offs applied during analysis. All projects stated that known anti-cancer and anti-leukaemia drugs made it onto the hit shortlist validating the specific approaches and model systems. The 25 hits listed by Erikson et al were iodoacetamide, NSC95397, brefeldin A, tetraethylthiuram disulphide, ouabain, idarubicin, ammonium pyrrolidinedithiocarbamate, mitoxantrone, quinacrine dihydrochloride, CGP-74514A, parthenolide, 2-(alpha-naphthoyl)-ethyltrimethylammonium iodide, emetine dihydrochloride hydrate, 2-chloroadenosine, L-703,606 oxalate, oligomycin A, VER-3323, podophyllotoxin, Bay 11-7085, colchicine, U-73122, cantharidin, diphenyleneiodonium chloride, wortmannin and spermidine trihydrochloride [60]. The authors went on to concentrate on quinacrine. Ouabain, one of their hits, also came up in the screen conducted by Laverdière and colleagues and was selected for further validation [68].

#### 2.2.2. Preclinical Validation

**Figure 2:**
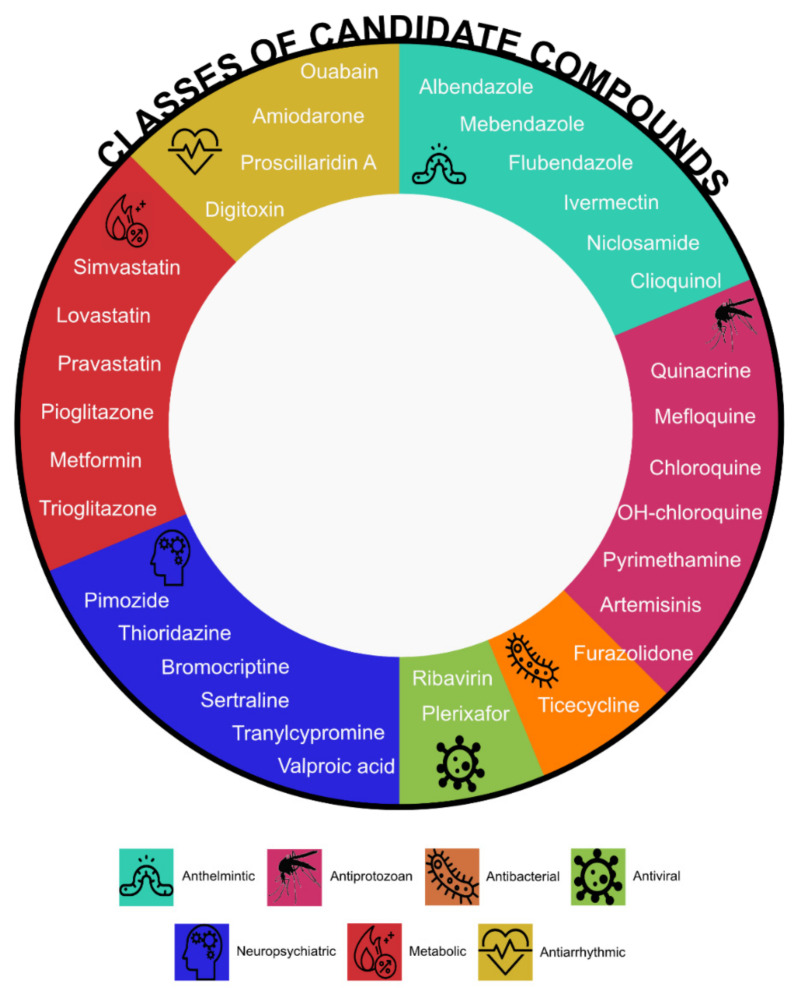
Candidate compounds to treat AML classified by original therapeutic indication.

All hits require careful validation in order to become promising candidates. Thirty-two compounds including antimicrobials (16), neuropsychiatric (6), metabolic (6) and antiarrhythmic (4) agents underwent preclinical validation in AML confirming the potential benefit for AML treatment (Figure 2).

There are six anthelmintic compounds with repurposing potential. Albendazole and mebendazole were identified through a chemical screen of selected compounds [61,64]. Both reduced colony formation in murine leukaemia cells but had negligible effects in normal murine bone marrow cells and caused reduced viability in THP-1 and OCI-AML3 human leukaemia cell lines. Ex vivo treatment of THP-1 pSLIEW cells transplanted into NSG mice showed decreased leukaemia burden and extended lifespan in the albendazole treated cohort compared to controls validating the results of the screen [64]. Flubendazole and ivermectin, identified through a small-scale chemical screen [51], induced cell death of leukaemic cell lines in vitro. Flubendazole delayed growth and reduced tumour weight in AML xenograft mouse models, whereas ivermectin induced intracellular chloride flux with a subsequent increase in reactive oxygen species and cell death in AML cells whilst sparing normal cells; it delayed tumour growth in three different leukaemia mouse models [52]. Niclosamide, which came up in a 2D chemical similarity analysis, appeared to be an ideal candidate for AML treatment due to its ability to inhibit NF-κB, mTOR, Wnt/ β-catenin and CREB signalling pathways [65,69,70,71]. Preclinical validation showed that it synergised with standard chemotherapy and increased survival of mice xenografted with AML cells [65]. Clioquinol, emerged as an inhibitor in the cyclin D2 transactivation screen, blocks the activity of proteasome and preferentially targets malignant AML. It decreased both tumour volume and weight without toxicity in three different xenograft AML mouse models [72].

Antiprotozoal (or antimalarial) compounds turned out to be a rich source of promising candidates. Screening over a thousand compounds from the LOPAC library (Sigma Aldrich) led to the identification of quinacrine (also known as mepacrine). It showed low toxicity in normal mononuclear cells and synergistic effect with cytarabine or azacytidine, in both in vitro and in vivo studies, resulting in a decrease of total number of circulating blast cells and increase in mice survival [60]. There is a growing interest in the use of chloroquine and hydroxychloroquine to treat cancer including AML [73]. Chloroquine was first fortuitously repurposed for the treatment of lupus erythematosus and rheumatoid arthritis due to improvement in soldiers with arthritis who took anti-malarial prophylaxis during World War II. In 1963, Hiraki and Kimura used chloroquine to treat tumours based on their “unique concept that damage of the stromal connective tissue will lead to the damage of tumour cell” [74]. Chloroquine synergised with phytochemicals and nonsteroidal anti-inflammatory drugs inhibiting the self-renewal of malignant stem or progenitor cells [75]; however, insufficient efficacy was observed in the in vivo studies [76]. Hydroxychloroquine induces cell death in cytarabine-resistant cells by blocking autophagy [77]. As cancer cells display high autophagic activity, inhibiting the process seems a valid therapeutic strategy. This may have been the reason of failure of the in vivo validation of chloroquine, which exerts its antileukaemic action in vitro through an autophagy-independent mechanism of action [76,78]. Sukhai and colleagues identified mefloquine by performing a screen of 100 on- and off-patent compounds. It selectively kills AML cells through the disruption of lysosomes at pharmacologically achievable concentrations and reduced tumour growth without significant toxicity in three AML mouse models [57]. Pyrimethamine is a drug used to treat malaria and toxoplasmosis found in a high throughput drug screen in the presence of ATRA. It is able to induce apoptosis and differentiation in human and murine AML cell lines without affecting normal cells both in vitro and in vivo [62]. Prompted by the success of the antimalarial category on the whole, nine antimalarial agents were tested on 10 cell lines to search for the most effective [79]. Artemisinins, including artesunate and dihydroartemisinin, showed selective anti-leukaemia against cells bearing MLL rearrangements and FLT3-ITD mutations. Although, the combination with cytarabine resulted in a synergistic effect in in vitro experiments, but did not prolong survival in vivo [79,80]. Furazolidone, an agent with antiprotozoal and antibacterial activity, was identified through a screen of 1200 FDA-approved compounds. Furazolidone has potent antiproliferative properties, induces apoptosis and morphological and phenotypical differentiation of AML cells [58].

The list of antimicrobials with repurposing potential also includes tigecycline, an on-patent antibacterial agent, used for the treatment of intra-abdominal or skin infections that was found in a chemical screen of ~300 compounds. Similarly to clioquinol and ivermectin, tigecycline selectively killed leukaemia cells and decreased tumour growth in a xenograft mouse model by blocking mitochondrial protein synthesis [53]. Repurposing of ribavirin, an antiviral compound used for the treatment of hepatitis C, stems from the discovery that it disrupts eIF4E eukaryotic translation initiation factor that is upregulated in human AML impeding differentiation and leading to transformation [81]. Ribavirin competes with mRNA for eIF4E binding and blocks oncogenic activity of eIF4 in vivo, as shown in mouse models and in AML patients [82]. AMD3100, also known as plerixafor, originally identified as an anti-HIV agent [83], is a CXCR4 antagonist. CXCR4-CXCL12 receptor-ligand axis normal and malignant bone marrow niche interactions, plays a role in AML formation and is an important therapeutic target as reviewed by us and others [84,85].

Anti-proliferative potential of psychotropic agents was known for over 20 years [86]. Six candidates for repurposing are neuropsychiatric therapeutics: three because they target important pathways and three identified through screening. Valproic acid (VPA), used to control convulsions in epilepsy, has been evaluated as an anti-cancer agent because of its histone deacetylase inhibiting properties. Histone deacetylases are often overexpressed in cancer cells and VPA induces differentiation or apoptosis in AML cells [87,88,89]. Antidepressant tranylcypromine is also an epigenetic modifier as it inhibits lysine-specific demethylase 1A (LSD1), an attractive epigenetic target for the treatment of AML. Leukaemic blast reprogramming rendered non-APL AML cells responsive to ATRA-induced differentiation [90]. Tranylcypromine treatment results in an increased myeloid differentiation in in vivo studies [91]. Antidepressant sertraline significantly decreases levels of TPT1, a target of tumour reversion, and kills cancer cells [92]. Sertraline exerts its antiproliferative activity inducing apoptosis and autophagy in both AML cell lines and primary human leukaemic cells [93]. Bromocriptine, a drug used in Parkinson’s disease, was identified through an in silico screen as a small molecule that induces cell differentiation and increases apoptosis in AML [63]. Bromocriptine holds promise for the treatment of high-risk myelodysplastic syndromes (MDS) and secondary AML as it showed selectivity for leukaemic cells as well as synergistic effect in combination with standard therapy [94]. Thioridazine is an agent for the treatment of psychotic disorders, withdrawn in 2005 due to its association with cardiac arrhythmias. It was since identified as a candidate for AML treatment through a discovery platform that determines differences between neoplastic and normal human pluripotent stem cells. Thioridazine induces differentiation of AML cells targeting cancer cells with no effects on normal human stem cells [56]. Pimozide, a psychotropic drug, demonstrated potential for being repurposed in AML treatment as it is able to inhibit the phosphorylation of the STAT5 transcription factor that is crucial for proliferation of leukaemic cells with FLT3-ITD mutation, resulting in cell death in vitro and in vivo [55].

Five agents that target cell metabolism are candidates for AML treatment. Amongst oral antidiabetics, application for metformin was found in oncology. Meta-analysis of 561,836 patients from 18 studies showed that metformin use was associated with a 27% reduction in any cancer risk [95]. Metformin exhibits efficacy against FLT3-ITD mutated AML cells when combined with sorafenib [96,97]. It synergises with cytarabine and exerts its anti-leukaemic effect through the inhibition of the mTORC1/P70S6K pathway [98]. Additionally, pioglitazone suppresses AML cell growth and, combined with standard chemotherapy, increased the remission rate in AML patients [99]. Moreover, biomodulatory treatment with azacytidine, ATRA and pioglitazone induced the development of normal cells [100,101]. A similar compound, trioglitazone, displayed cell-non-autonomous anti-leukaemia activity. LSCs plated on stroma and pre-treated with it did not form cobblestone areas highlighting the possibility of leukaemia elimination through niche-interaction modification [59]. Statins are under consideration as antileukaemic agents as their intake diminished the risk of developing haematological diseases in a meta-analysis of 14 observational studies [102]. An increase in 3-hydroxy-3-methylglutaryl-coenzyme A (HMG-CoA) reductase enzyme quantity and its catalytic activity in AML was an early observation [103]. Statins are effective inhibitors of the HMG-CoA reductase hence their utility in AML. Simvastatin, pravastatin and lovastatin showed an inhibitory effect on leukaemic progenitors [104]. In addition, lovastatin inhibited cobblestone area formation of LSCs derived from AML patients through HMG-CoA reductase inhibition. No inhibition of normal haematopoietic stem and progenitor cell cobblestone area formation was observed under the same conditions. Lovastatin showed activity in vivo in syngenic bone marrow transplantation AML models [59].

Amiodarone, digitoxin, ouabain and proscillaridin A are medications for heart conditions and are under evaluation as anti-leukaemia agents. Amiodarone is a potassium channel blocker approved to treat life-threatening ventricular arrhythmias. In vitro studies showed that the combination of amiodarone with ABT-263, an inhibitor of anti-apoptotic BCL2-like protein, increased apoptosis in human leukaemia cell lines [66]. Digitoxin and ouabain are cardiac glycosides sometimes used in place of digoxin for the treatment of heart failure and arrhythmia. An in silico analysis followed by in vitro leukaemic stem cells assay showed that both digitoxin and ouabain effectively eliminated human AML stem cells [68]. In addition, an unbiased transcriptomic approach demonstrated that cardiac glycosides inhibit general protein synthesis; unfortunately, this effect occurs in both cancer and normal cell lines. Thus, results from studies that demonstrated anti-cancer activity of digitoxin (or in general cardiac glycosides) that used xenograft mouse models necessitate re-examination [105] as murine cells are more resistant to cardiac glycosides, and mice tolerate high levels of cardiac glycosides, than humans. Proscillaridin A targets MYC-overexpressing leukaemias through global loss of lysine acetylation with IC50 for LSCs half that of bulk human AML 8227 cells [106].

In summary, of 32 compounds considered for repurposing in AML, 27 were successfully validated in the preclinical setting aimed at leukaemia elimination. A different aspect of AML treatment was addressed by another repurposing study. Anti-AML chemotherapy affects rapidly cycling normal multipotent and lineage-committed progenitors causing anaemia, neutropoenia and thrombocytopaenia. Quizartinib, an FLT3 inhibitor frequently mutated in AML, induces the quiescence of normal murine multipotent progenitors conferring a significant protection to mice treated with chemotherapy without affecting the elimination of FLT3-independent tumours by cytotoxic drugs [107].

#### 2.2.3. Clinical Trials

Of the 32 compounds deemed to possess repurposing potential, 13 are under investigation in clinical trials according to data entered into the clinicaltrials.gov database, the main source of information for this section of the review (Table 2). Clioquinol, tigecycline and ribavirin were tested as monotherapy, others in combination with known anti-AML agents.

Five antimicrobials entered into the clinical trial phase. The tolerance and biological activity of oral clioquinol were evaluated in 11 patients with relapsed or refractory haematological malignancy is reported (NCT00963495). Although safe up to a dose of 1600 mg twice-daily, no useful clinical responses were observed, probably, due to low intracellular concentration of the drug resulting in a minimal proteasome inhibition [108]. University of Pittsburgh Cancer Institute (Pittsburgh, PA, USA) initiated a phase I clinical trial (NCT02631252) to establish the safest effective dose of the combination of mitoxantrone (an FDA-approved drug for the treatment of AML) and etoposide (not FDA-approved for AML, but commonly used in AML) with hydroxychloroquine; the trial was terminated due to inability to accrue (clinicaltrials.gov; data accessed: 14 March 2020).

The safety of tigecycline was evaluated via an open-label phase I clinical trial in 27 patients (median age 70) with relapsed and refractory AML comprising seven patients with poor risk cytogenetics (NCT01332786). Analogously to clioquinol, the trial demonstrated a favourable safety profile of tigecycline at doses of 300 mg/day and lack of response to therapy leading to the closure of the trial. The inefficacy of tigecycline may be due to its short half-life [110].

Ribavirin has undergone four clinical trials: two completed, one with unknown status and one still recruiting. The first phase II clinical trial (NCT00559091) to assess ribavirin’s efficacy against AML M4 and M5, which enrolled 13 patients, started in 2007, three years after the emergence of preclinical evidence for ribavirin activity against AML [111]. CRs and PRs were observed after 30 days of treatment, albeit responders relapsed shortly after acquiring resistance. All patients in the trial had elevated levels of eIF4E: the response was linked to decreased eIF4E mRNA levels, with response loss to the re-entry of eIF4E into the nucleus [112]. Partial success of this trial led to the opening of a new study to investigate the effect of ribavirin in combination with low-doses of cytarabine (NCT01056523) on 29 recruited patients (median age 65), unfit for intensive chemotherapy or with relapsed/refractory AML and elevated eIF4E. Two patients achieved temporary CR, one-PR and two-blast responses. The combination was well tolerated and seemed better than monotherapy [127]. Short duration of responses was blamed on increased levels of the sonic hedgehog transcription factor Gli1, as it inhibits the interaction between ribavirin and eIF4E. Thus, ribavirin was combined with a Gli1 inhibitor (i.e., vismodegib) in a new trial (NCT02073838) of which no results are available (clinicaltrials.gov; data accessed: 14 March 2020). One more ongoing trial will assess the efficacy of combining decitabine, a drug used in patients with MDS, with rapamycin or ribavirin in high risk AML patients (NCT02109744); a recruiting status is reported (data accessed: 14 March 2020).

Plerixafor in combination with different agents underwent nine clinical trials for the treatment of AML patients as reported at clinicaltrials.gov. The first phase I/II clinical trial (NCT00512252) to evaluate the safety and efficacy of plerixafor in combination with mitoxantrone, etoposide and cytarabine (MEC), started in 2007 and demonstrated that plerixafor was safe at a dose of 0.24 mg/kg/day; 21/56 patients (median age 52) achieved CR and complete remission with incomplete blood count recovery (CRi) with a median OS of 8.2 months and relapse-free survival (RFS) of 9.0 months. Plerixafor mobilised leukemic blasts into circulation abrogating protective signalling from the niche [114]. The same research group conducted another phase I/II trial adding granulocyte-colony stimulating factor (G-CSF) to plerixafor/MEC treatment in a group of 35 patients (median age 56; NCT00906945), successfully triplicating its dose. Unfortunately, the number of patients who achieved CR/CRi was less than the number required by the study design to proceed with phase II and the study was terminated [128]. The third trial to evaluate the efficacy of plerixafor/MEC was terminated due to sponsor’s support withdrawal (NCT01027923). Plerixafor was then combined with standard chemotherapy +/−G-CSF (NCT00990054 and NCT01455025). There are no results available for NCT00990054 initiated in 2009, whereas NCT01455025 was closed due to slow recruitment. In 2010, a phase I/II trial conducted in elderly AML patients established that plerixafor was safe up to 0.4 mg/kg in combination with a fixed dose of clofarabine; the study was closed prior to phase II completion due to slow accrual (NCT01160354). Later that year, the safety of plerixafor in combination with sorafenib and G-CSF was investigated (NCT00943943); no results are available. In 2011, AML18 pilot investigated the effect of quizartinib in combination with plerixafor or ganetespib with standard chemotherapy in elderly AML or high-risk MDS patients (NCT01236144). Although 44 cases were enrolled in the plerixafor arm, the drug manufacturer stopped it [129]. In the same year, the efficacy of plerixafor in combination with decitabine as induction and post-remission therapy for elderly patients with AML was evaluated in 55 patients recording CR/CRi in 49% of patients (NCT01352650). The study demonstrated that CD25 expression was associated with poor outcome and chemotherapy resistance in newly diagnosed older or unfit AML patients treated with decitabine and plerixaflor [130]. Finally, a PLERIFLAG phase II trial, which enrolled 41 patients (median age 52), established the recommended dose of plerixafor plus fludarabine, idarubicin, cytarabine and G-CSF and obtained 48% CR/CRi in early-relapsed or primary refractory AML with median OS and disease-free survival (DFS) of 9.9 and 13 months, respectively (NCT01435343) [131].

Twelve clinical trials of VPA are registered in the clinicaltrials.gov database, of which nine are completed. VPA has been tested in combination with decitabine, ATRA, theophylline, cytarabine and 5-azacytidine. In 2001, the first in vitro evidence of anticancer activity of VPA emerged [89]. The first trial of VPA started in January 2004 and evaluated VPA cotreatment with decitabine in relapsed/refractory leukaemia or MDS (NCT00075010) enrolling 54 patients. VPA doses of 50 mg/kg daily was found to be safe. Ten patients achieved CR and two-CR with incomplete platelet recovery (CRp). The median remission and OS in responders lasted 7.2 and 15.3 months, respectively. Combination treatment was associated with transient DNA hypomethylation and global histone H3 and H4 acetylation resulting in p15 reactivation [132]. Later that year, another study assessed low-dose decitabine combined with VPA for the treatment of patients affected by refractory or relapsed AML, chronic lymphocytic leukaemia or with small lymphocytic lymphoma (NCT00079378). Clinical responses were similar in decitabine alone or decitabine plus VPA groups, but the combination resulted in encephalopathy at relatively low doses [115]. Two years later, a phase II clinical trial re-evaluated the decitabine plus VPA combination versus decitabine alone in MDS and AML (NCT00414310) on a cohort of 150 patients (median age 69). In the combination group, 39/79 (49.3%; CR, PR or CRi) patients responded compared to 28/70 (40%; CR, CRi) in the decitabine group; the response was more durable in the decitabine only group (12.9 versus 6.3 months, respectively); OS rate was similar in the two groups. Encephalopathy during combination treatment occurred in <2% of patients. Results obtained in a non-randomized phase II trial conducted in elderly non-fit AML patients treated with decitabine alone or combined with ATRA [133], prompted phase II clinical trials to assess the combination of decitabine with VPA, with ATRA or with both drugs in 200 elderly (median age 76) AML patients (NCT00867672). While the addition of ATRA to decitabine resulted in a higher overall response rate (ORR) without additional toxicity and in OS extension, also in patients with poor cytogenetics, the addition of VPA did not affect the ORR or OS [134]. By contrast, VPA was successfully combined with ATRA and theophylline or with ATRA and low-doses of cytarabine (NCT00175812, NCT00995332). The combination of short-term ATRA, low-dose VPA and theophylline was safe even in elderly patients resulting in disease stabilization and blood cell count normalisation (transient decrease in circulating blasts) [135]. The combination of VPA, ATRA and low-dose cytarabine in a group of 36 patients (median age 77) resulted most frequently in increased and stabilised platelet counts, whereas only two patients achieved CR [136]. A randomized phase III study (NCT00151255) to evaluate the safety and efficacy of VPA combined with induction therapy and ATRA reported no clinical improvement in the VPA group compared to standard treatment. However, RFS was significantly higher in VPA than in standard-treatment group. Patients with NPM mutation responded better to the treatment [137]. No clinical efficacy after a maximum of four courses was reported from a phase II clinical study evaluating 5-azacytidine plus VPA or low-doses of cytarabine in 11 elderly patients with untreated AML or high risk MDS (NCT00382590), whilst the combination of 5-azacytidine, VPA and ATRA was successful. Two phase II clinical trials (NCT00326170, NCT00339196) hypothesised that the combination of a hypomethylating agent (5-azacytidine) with an HDAC inhibitor (VPA) would revert ATRA resistance. The combination was considered safe; ORR was 42%: 12/53 (22.6%) patients achieved CR including those with poor-risk cytogenetics. The therapy was associated with significant DNA hypomethylation and induction of histone acetylation [138]. The results were confirmed by the NCT00339196 trial in patients with high-risk AML or MDS treated with the same combination. Early platelet response and demethylation of the FZD9, ALOX12, HPN and CALCA genes during the therapy were associated with clinical response [139]. Currently, two clinical trials for VPA are still recruiting and with no preliminary results available. One is investigating the efficacy of 5-azacytidine and VPA as post allogeneic transplant treatment for high-risk AML and MDS (NCT02124174); the other the effect of combined 5-azacytidine, VPA, hydroxyurea and ultimately donor leukocyte infusion for the treatment of AML relapse after allotransplantation (NCT01369368).

A trial to assess the safety of thioridazine in combination with cytarabine in relapsed or refractory AML or patients in advanced-stage MDS (NCT02096289) recruited 13 patients (median age >65 years). Leukaemic burden decreased in 8/11 patients during initial thioridazine treatment and continued to decline when cytarabine was added. Blasts increased when thioridazine was stopped, suggesting that chronic administration is required for their suppression; however, thioridazine intake is limited by its cardiotoxicity and neurological events. Thus, modifications are needed to improve tolerability [116]. Currently, a phase I trial assessing the benefit of sertraline in combination with cytarabine in relapsed and refractory AML is recruiting (NCT02891278; data accessed: 14 March 2020). Three phase I clinical trials to evaluate the safety and efficacy of tranylcypromine and ATRA treatment for adult patients with AML and MDS are ongoing (NCT02261779, NCT02273102, NCT02717884) with no preliminary results posted (clinicaltrials.gov, data accessed: 14 March 2020).

Metformin’s efficacy against AML cells was established in 2010 by Scotland and colleagues [118]. Five years later, a phase I clinical trial commenced to evaluate metformin in combination with standard treatment and closed a year later in January 2016 due to slow accrual (NCT01849276). A trial called AML-ViVA combining ATRA, pioglitazone and low-dose 5-azacytidine compared to standard-dose 5-azacytidine (NCT02942758) was initiated following the success of the ViVA off-label therapeutic regimen in five patients with refractory AML [100]. The trial’s preliminary results showed that the combination was safe with no dose-limiting toxicity in 10 patients (median age 67). Three patients achieved CR and one patient remained stable 14 months post-treatment [121]. Another trial evaluated the safety and efficacy of pioglitazone in combination with standard therapy randomising 40 newly diagnosed AML patients into two groups: cytarabine plus daunorubicin +/− pioglitazone. The CR rate was greater in the pioglitazone group compared to control subjects [99]. 

Several trials were carried out to assess the potential of statins in AML treatment. Ten years since the first in vitro evidence of pravastatin utility against AML, Kornblau et al hypothesised that the pravastatin added to standard therapy may block adaptive cholesterol responses in AML blasts improving therapeutic efficacy and put this theory to a phase I trial. The combination was safe and efficient: 11/15 newly diagnosed AML patients including those with poor cytogenetics and 9/22 salvage patients achieved CR or CRp [122]. The SWOG phase II study ensued to evaluate the effect of the pravastatin, idarubicin and cytarabine combination in relapsed AML patients (NCT00840177). The response rate was 75% and the median OS was 12 months [140]. The study closed the accrual after meeting criteria for a positive study, but was later amended to comprise poor-risk patients recording a response rate of 30%, also in patients with FLT3 mutations and ones that underwent hematopoietic stem cell transplantation; the median OS was 27.1 months [141]. The SWOG results incited Shadam et al, to ascertain if pravastatin combined with idarubicin and cytarabine safely increased the CR rates without minimal residual disease and with a rapid count recovery (defined by the authors as “good CRs”) in adults affected by newly-diagnosed AML (NCT01831232). Half (12/24) of the cohort achieved good CR, but four relapsed after a median of 16 weeks and two died from multi-organ failure. Based on these results, the authors did not recommend the combination regimen for phase III trial in untreated AML and high-risk MDS [142]. Another phase I/II trial assessed the efficiency of etoposide and mitoxantrone administered with cyclosporine and pravastatin to treat patients with relapsed or refractory AML (NCT01342887), finding the combination excessively toxic and the efficacy not acceptable in this subset of patients [143]. The study to test the safety and efficiency of adding lovastatin to the standard chemotherapy was terminated due to slow accrual (NCT00583102). Finally, a pilot trial of atorvastatin in tumour protein 53 (p53)-mutated and p53 wild-type malignancies including solid tumours and AML is actively recruiting (NCT03560882; data accessed: 14 March 2020).

A trial of digoxin (and not digitoxin) combined with decitabine for the treatment of AML and MDS was started in 2017 and then terminated due to slow accrual (NCT03113071). The remaining candidate compounds never entered into clinical trials.

## 3. Where Are We Now?

**Figure 3:**
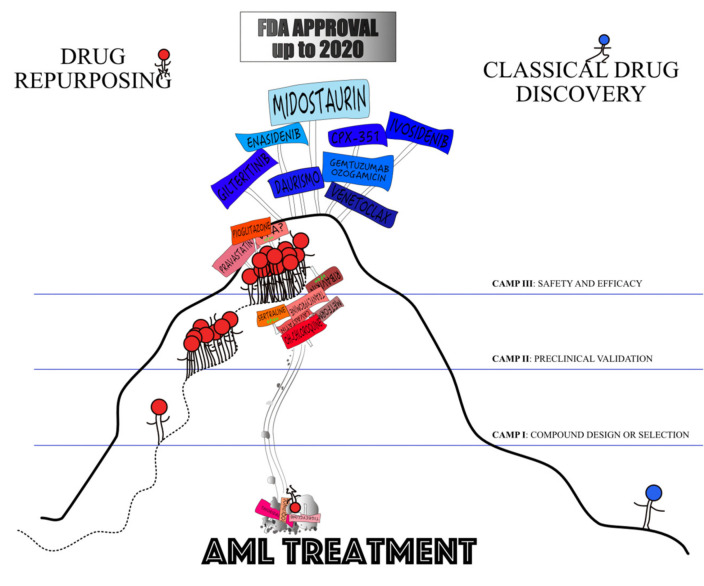
Drug discovery is like climbing a mountain. Repurposing partially follows beaten tracks whilst de novo development carves out new paths for itself. So far only agents developed de novo made it to obtaining the FDA approval.

None of the candidate compounds have so-far obtained FDA or EMA approval and, therefore, have not been introduced into AML treatment (Figure 3). The clinical validation has been disappointing for the vast majority of the 13 compounds that made it into clinical trials. The promising combinations resulting in robust responses included VPA with 5-azacytidine and ATRA, plerixafor with decitabine or plus fludarabine, idarubicin, cytarabine and G-CSF, pioglitazone with low-dose 5-azacytidine or with standard treatment and pravastatin with standard treatment. VPA with theophylline or low-dose cytarabine, instead, may be useful in un-fit patients to stabilise the disease.

The main issue encountered was the lack of efficacy stalling the agents in phase I and II clinical trials. Clioquinol, tigecycline, ribavirin, VPA in some combinations elicited no, little or transient responses. Ribavirin as monotherapy showed an initial short-term response; it worked better in combination with cytarabine, but the latter altered its absorption diminishing the activity [112,127]. Diverse responses were obtained with VPA and pravastatin in some combinations tested. While encouraging results were obtained for the combination of VPA with ATRA and theophylline [135] or with cytarabine [136] or with 5-azacytidine [138,139], conflicting data come out of clinical trials to assess the efficacy of VPA and decitabine. Only one small trial reported the advantage of adding VPA to decitabine. Two others showed similar response rates with transient effect in decitabine-alone and decitabine plus VPA groups. The addition of VPA in ATRA plus decitabine or in the induction therapy setting did not increase OS [115,132,133,134,137]. The combination of pravastatin with cytarabine and idarubicin was successful in two out of three clinical trials for the treatment of newly-diagnosed and relapsed AML patients [122,140,141,142].

Toxicity of the repurposed drugs is an aspect not to be underrated. The combination of VPA and decitabine is associated with marked and unacceptable VPA-dependent encephalopathy. Pravastatin together with idarubicin and cytarabine resulted in multi-organ failure. Additionally, pravastatin with etoposide, mitoxantrone and cyclosporine and thioridazine combined with cytarabine had adverse effects [116,143].

Last but not least, slow accrual led to a premature closure of six clinical trials (i.e., hydroxychloroquine, plerixafor (two trials), metformin, lovastatin and digoxin); maybe extra funding is necessary for trial success.

When reviewing drug repurposing in cancer, one must not forget to mention the Repurposing Drugs in Oncology (ReDO) project. It draws attention to the new potential oncological uses of well-known and well-defined non-cancer drugs through an accurate analysis of the literature on the premise that most drugs act on more than one target within a cell. The ReDO project identified 310 non-cancer agents with “hard” repurposing potential supported by preclinical and clinical data. Most of them (85%) were off-patent compounds and at least 67% had data on patients such as case reports, observational studies or clinical trials supporting their anticancer effects (ReDo database accessed: 7 March 2020) [144,145,146]. With the exception of clioquinol, furazolidone, plerixafor, tranylcypromine, trioglitazone and proscillaridin A, all candidates for AML treatment are included in the ReDO project.

## 4. Does the Promise Hold?

Is the drug repurposing strategy delivering new therapeutic options in AML or is it not? Every scientific publication concerning the re-tasking approach lists a number of statements that have become dogmas, but are they incontrovertibly true?

- Dogma 1: Drug repurposing saves time

It took a median of 4.8 (0–13) years from the initial report of compounds’ validation as potential anti-AML agents to their entry into a clinical trial (Table 2) with tigecycline being tested the fastest and pioglitazone the slowest. We compared the development times of seven de novo agents recently approved for AML treatment (i.e., midostaurin, gilteritinib, enasidenib, ivosidenib, venetoclax, daurismo and CPX-351; Table 3) with corresponding times for the repurposed compounds evaluated in AML. The de novo drug discovery process required a median of 0.4 (0–3) years to proceed from preclinical to clinical research. Then, a median of seven years (from 4 to 12) elapsed from the first clinical trial to FDA approval for a total of 10.4 years (Table 3). Paradoxically, if all repurposed drugs were to be approved in 2020, a median of 8.4 years would have passed between the first clinical trial and marketing approval for a total of 13 years from preclinical validation to FDA approval. In AML, drug repurposing approaches have failed to speed up the development process.

- Dogma 2: Phase I clinical trials can be skipped.

Eight out of fifteen initial clinical trials were phase I trials, as agents were administered at higher doses than for their original therapeutic indication, to assess the safety of combinations even at lower doses than standard dosing or because of a novel route of administration (Table 4).

New safety evaluation as primary trial objective was needed to establish dose-limiting toxicity (DLT) and the maximum tolerated dose (MTD) of oral clioquinol that is normally used topically.

Doses different to the original are often required to target new molecular pathways of repurposed agents and their safety has to be re-ascertained. In AML, hydroxychloroquine was given for 21 days at the dose of 600–1400 mg/day against a total of 2000 mg divided over 48 h in acute malaria or 400 mg per week in malaria prevention. Tigecycline required re-testing because doses up to seven times higher than the standard dose of 50 mg/day were given in 3-week cycles compared to its 14-day cycle as antibiotic. The doses of sertraline and pioglitazone were slightly higher than standard (max. 250 mg/day in trial versus max. 200 mg/day in depression, 45 mg/day in trial versus max. 30 mg/day in diabetes). Pravastatin and lovastatin, instead, were used at significantly higher doses (up to 1680 mg/day) than when used in hypercholesterolaemia and similar therapeutic indications (up to 80 mg/day).

Phase I clinical trials were performed for the combinations involving hydroxychloroquine, plerixafor, thioridazine, sertraline, metformin, pravastatin and atorvastatin. The trial to assess the use of metformin to treat AML specifically sought to determine DLT and MTD of metformin in combination with cytarabine as a primary objective in patients with relapsed/refractory AML; metformin was administrated at its standard dose for the treatment of diabetes type II (standard dosage taken from drugs.com database).

- Dogma 3: Repurposed drugs are safe as their toxicity profile is known

Three out of fifteen (20%) trials were stopped because of toxicity.

Does the promise hold? Hardly. A very recent review on drug repurposing in AML asked if it were a futile pursuit and responded by saying that even a failed attempt furthers our knowledge of AML biology [174].

What needs to be done to increase the chances of success? One can think of improvements at the level of screening and preclinical validation to identify more robust candidates. Thus, we need better tools to screen. Numerosity of compounds in libraries has brought about the demand for increased throughput leading to the miniaturisation of screening formats and the introduction of droplet-based-, continuous-flow-, digital- and paper-microfluidics (reviewed in [175,176]). The integration of microfluidics with artificial intelligence-driven data collection and analysis systems will be the future. There is room for improvement of disease models. For example, a screen performed in a haematopoietic niche-like microenvironment identified a subset of compounds that were not hits using traditional cell line models evidencing the importance of appropriate model systems [59], as targeting LSCs is more relevant for AML eradication. More sophisticated read-out systems are called for too as vitality assays do not seem good enough—some compounds kill because they are toxic. All data from screening should be available for others to mine and it should become a custom to publish the entire shortlist. The identification of the same compound as a hit in more than one screening effort may be useful to decide whether to invest in it. More attention should be paid to the genetic, epigenetic heterogeneity of AML to find mutation-specific therapies. Detailed molecular profiling of patients has to be done in parallel with the assessment of response, be it patient’s bone marrow sample or the individual. Recently, Chiu and colleagues developed a microfluidics-based chip for nucleic acid manipulation and analysis in single cells. Such chip could be adopted to detect genetic mutations responsible for resistance and relapse of individual AML patients for personalised medicine approaches [177].

The limited scale and number of studies suggests that money is a real problem when repurposing off-patent compounds. More small clinical trials should be conducted as they are less expensive and quicker. Not-for-profit organization such as universities, hospitals, foundations, research institutes and governments could fund these trials without a contribution from pharmaceutical industry. Recently, the Orphan Drug Act, incentivised rules have been proposed to overcome all these issues for manufacturers licensing generic drugs [2,178]. AML falls into the category of orphan diseases based on its frequency [4]. Importantly, off-label prescription of a repurposed compound should be avoided to force legal authorization, reimbursement and clinical adoption of such agents [178,179].

It transpires from the analysis conducted that there may be a kind of distrust towards the candidates to be repurposed and, as a consequence, the trials fail to accrue sufficient numbers. In one case, the move of the principal investigator was a reason for the trial’s closure (NCT00583102). The wariness of new therapeutic options emerging from repurposing may be caused by the full awareness of the clinicians involved that AML is a serious neoplastic disease requiring an aggressive and effective treatment and, therefore, hardly anybody is prepared to try something “new”, but not entirely new. The disease progresses too fast to afford the luxury of calmly testing a potential candidate and switching back to something else should the drug fail to work. The haematologists probably do not want to renounce the “certainties” of standard therapy and exchange them for the unknown efficiency of novel options. Clearly, de novo drug discovery gives no guarantees of success either, but studies are sponsored by the big pharma and are somehow more accepted by clinicians and patients alike.

## 5. Conclusions

Taken together, it is hard to be enthusiastic about drug repurposing in AML. It identified a host of candidates for the treatment of AML through diverse wet and in silico approaches from antimicrobial, neuropsychiatric, metabolic and cardiac medication categories. Although most candidates belonged to antimicrobials, all but one was unsuccessful in clinical setting. The results obtained for plerixafor with decitabine or plus fludarabine, idarubicin, cytarabine and G-CSF, VPA with 5-azacytidine and pioglitazone with low-dose 5-azacytidine or with standard treatment are the most promising, but the bottom line is that no “old” drug has so far been effectively repurposed and none is close to being approved. The development times have been long and clinical trials were troubled by lack of efficacy, toxicity and slow accrual.

Traditionally, anti-AML treatment was based on a relatively small number of molecules with anti-proliferative potential used in all diagnoses. The complexity of molecular pathways deregulated or highjacked by leukaemia and the heterogeneity of disease phenotypes call for specific agents that will target vulnerabilities of individual diseases. Compound libraries are a source of infinite therapeutic options that should be continuously explored although there is no easy way to go about it.

## Figures and Tables

**Table 1:** Screening projects to identify candidate compounds for repurposing in AML.

Year	Screen	Library (Compound Number)	Model System	Readout	Concentration	Assay Time	Hits (%)	Candidate	Reference
2007	Cyclin D2 transactivation inhibitor screen	Prestwick (1120) and LOPAC (1280)	NIH3T3 expressing c-Maf and the cyclin D2 promoter-driven luciferase reporter	Luminescence and MTS reduction viability assay	5 µM	20 h	39 (1.6)	Clioquinol	[49,50]
2010	Chemical screen	Custom * (110)	OCI-AML2, HL60, KG1a	MTS reduction viability assay	5–50 µM	72 h	Several	**Flubendazole**	[51]
2010	Chemical screen	Custom *^,$^ (100)	OCI-AML2, HL60, KG1a	MTS reduction viability assay	5–50 µM	72 h	NR	**Ivermectin**	[52]
2011	Chemical screen	Custom * (312)	TEX and M9-ENL1 with LSC features	MTS reduction viability assay	1–10 µM	72 h	5 (1.6)	**Tigecycline**	[53]
2012	STAT5 transcriptional activity inhibitor screen	Prestwick (1120)	U3A cell line expressing STAT5 promoter-driven luciferase reporter + cytokine induction	Luminescence	0–10 µM	2 + 6 h	NR	**Pimozide**	[54,55]
2012	Chemical screen	NIH Clinical Collection (446) and Canadian Compound Collection (144)	Neoplastic and normal hPSCs transduced with EOS-GFP reporter indicative of Oct4 and Sox2 expression levels	Differentiation induction assessment by automated microscopy	10 µM	72 h	11 (1.8)	**Thioridazine**	[56]
	Secondary high content analysis	11					3 (27)		
2013	Chemical screen	Custom *^,$^ (100)	OCI-AML2, HL60, KG1a	MTS reduction viability assay	5–50 µM	72 h	Several	**Mefloquine**	[57]
2013	Chemical screen	Prestwick (1120)	Primary murine BM cells transformed with *AML1-ETO*, cells from second plating	Methylcellulose colony formation assay	50 µM	5–7 days	95 (8.4)	Furazolidone	[58]
2013	Chemical screen	Broad Institute Compound Collection (14718) **	Primary murine LSCs from quaternary *MLL-AF9* AML grown on primary or OP9 stroma cells mimicking the niche	Leukemic cobblestone area-forming cell assay	5 µM	5 days	415 (2.8)	**Lovastatin** **Trioglitazone**	[59]
2015	Chemical screen	LOPAC (1280)	Primary AML and PBMNC samples (4+4; also, ALL and CLL)	Fluorometric Microculture Cytotoxicity Assay	10 µM	72 h	25 (1.9)	**Quinacrine**	[60]
2016	Chemical screen	Screen Well FDA-approved drugs (760) ^	Primary murine cell lines representative of AML and MLL	<50% viability	NR	NR	38 (5)	**Mebendazole**	[61]
2016	Chemical screen in the presence of ATRA	Biomol (36), MicroSource (1214), Prestwick (1120), Sigma (885)	MN1-transformed murine bone marrow progenitors, known to be resistant to ATRA	Alamar blue viability assay, <80% viability	2.5 µM + 1 µM ATRA	45 h	117 (3.2)	**Pyrimethamine**	[62]
2016	In silico: CMap (Build 1.0)	164 perturbagens	Published expression profile of the HL60 cell line treated with PMA; CMap database	PMA-differentiation signature crossed with CMap; *p* value <0.05 and a connectivity score >0.75 in HL60 at a concentration <10 µM	N/A	N/A	NR	**Bromocriptine**	[63]
2017	Chemical screen	Screen Well FDA-approved drugs (760) ^	Primary murine cell lines representative of AML with a t(9;11) (*MLL-AF9* translocation) and AML with a normal karyotype (*HOXA9-Meis 1* -driven).	<50% viability	NR	NR	38 (5)	**Albendazole**	[64]
2018	In silico: 2D chemical similarity analysis	NR	N/A	Structural similarity to XX-650-23 CREB inhibitor	N/A	N/A	NR	**Niclosamide**	[65]
2018	Chemical screen in the presence of ABT-737 BCL2 inhibitor.	LOPAC (1280)	RPMI 8226, U937, HL60	CellTiterGlo viability assay	1.8, 9, 45 µM + IC30 or IC70 of ABT-737	48 h	NR	**Amiodarone**	[66,67]
2018	In silico: CMap (Build 2.0)	Small bioactive molecules and tools (1310)	CMap database	First query with two LSC signatures to identify compounds that inhibit LSC gene expression programmes. Second query of the CMap to exclude compounds that inhibit HSCs	N/A	N/A	151 (11.5)	**Digitoxin** **Ouabain**	[68]
	Secondary chemical screen	84/151 compounds from the in silico screen.	AML 8227	Phenotype screen by flow cytometry	2.5, 5, 10 µM	6 days	48		

Legend: * on- and off-patent drugs including antimicrobials and metabolic regulators; ** chemically diverse commercially available and internally synthesized libraries, including ~1.920 known bioactive molecules; ^$,^^: the same screen; N/A: not applicable; NR: not reported; HSC: haemopoietic stem cell; LSC: leukaemic stem cell; MTS: 3-(4,5-dimethylthiazol-2-yl)-5-(3-carboxymethoxyphenyl)-2-(4-sulfophenyl)-2H-tetrazolium); AML: acute myeloid leukaemia; ALL: acute lymphoblastic leukaemia; CLL: chronic lymphocytic leukaemia; BM: bone marrow; FDA: Food and Drug Administration; LOPAC: Library of Pharmacologically Active Compounds; NIH: National Institutes of Health; PBMNC: peripheral blood mononuclear cells; CMap: Connectivity map (Broad Institute); ATRA: all-trans retinoic acid. ReDO (Repurposing Drugs in Oncology project) compounds are indicated in bold.

**Table 2:** Starting year of first clinical trials for repurposed candidates.

Drug Group	Candidate Compound		First Preclinical Study (Year)	First Clinical Trial (Year)	NCT Number	Status	Phase	Years between Preclinical and Clinical Study
Anti-microbial	Clioquinol		2007 [50]	2009 [108]	NCT00963495	Terminated	I	2
	Hydroxychloroquine		2015 [77]	2016 [109]	NCT02631252	Terminated	I	1
	Tigecycline		2011 [53]	2011 [110]	NCT01332786	Completed	I	0
	Ribavirin		2004 [111]	2007 [112]	NCT00559091	Completed	II	3
	Plerixafor		2004 [113]	2007 [114]	NCT00512252	Completed	I/II	3
Neuro-psychiatric	Valproic acid		2001 [89]	2004 [115]	NCT00075010	Completed	I/II	3
	Thioridazine		2012 [56]	2014 [116]	NCT02096289	Completed	I	2
	Sertraline		2004 [116]	2016 [117]	NCT02891278	Recruiting	I	12
	Tranylcypromine		2012 [90]	2014 [90]	NCT02261779	Unknown	I/II	2
Metabolic	Metformin		2010 [118]	2015 [119]	NCT01849276	Terminated	I	5
	Pioglitazone		2004 [120]	2017 [121]	NCT02942758	Recruiting	II	13
	Statins	Pravastatin	1997 [104]	2007 [122]	NA	Completed	I	10
		Lovastatin	1997 [104]	2001 [123]	NCT00583102	Terminated	I/II	4
		Atorvastatin	2007 [124]	2018 [124]	NCT03560882	Recruiting	I	11
Cardiac	Digoxin		2016 [125]	2017 [126]	NCT03113071	Terminated	I/II	1

NA – not available.

**Table 3:** Development times of FDA-approved de novo agents for AML treatment.

Compound	Year of Development	First Preclinical Study (Publication Year)	First Clinical Trial (Starting Year)	FDA Approval	Years Between Development and Preclinical Studies	Years Between Preclinical and Clinical Studies	Years Between Clinical Trial and FDA Approval	Total Drug Discovery Time
Midostaurin	1986 [147]	2002 [148]	2005 [149]	2017 [150]	16	3	12	31
Gilteritinib	2013 *	2014 [151]	2013 [152]	2018 [153]	1	N/A	5	5
Enasidenib	2010 ^§^ [154]	2013 [155]	2013 [156]	2017 [157]	3	0	4	7
Ivosidenib	2012 ^^^ [158,159]	2014 [160]	2014 [161]	2018 [162]	2	0	4	6
Venetoclax	2013 [163]	2014 [164]	2013 [165]	2018 [166]	1	N/A	5	6
Daurismo	2011 [167]	2016 [168]	2010 [169]	2018 [170]	5	N/A	8	7
CPX-351	2006 [171]	2006 [171]	2006 [172]	2017 [173]	0	0	11	11
**Median**					**4**	**0.4**	**7**	**10.4**

* year of first clinical trial given as information on development year is not available; § year of the start of a strategic collaboration between Agios and Celgene to develop new therapeutics targeting cancer metabolism; ^ year of optimization of AG-5198 developed in 2012.

**Table 4:** New and old dosing of repurposed compounds in AML.

Compound	First Clinical Trial in AML	Original Indication
Clioquinol	Oral administration	Topical application
OH-chloroquine	600–1400 mg/day for 21 days	Acute malaria: 800 mg +400 mg (6h) + 400 mg (24h) + 400 mg (48h); Malaria prevention: 400 mg/week
Tigecycline	50–350 mg/day (7 levels, 3-week cycles)	Initially, 100 mg, then 50 mg/day for 21 days
Ribavirin	1000 mg/day	2000 mg/day
Plerixafor	Escalation 0.08–0.24 mg/kg/day (= ~6–18 mg/day)	Max. 80 mg/day
VPA	20 mg/kg/day for 10 days	10–15/kg/day, max. dose 60 mg/kg/day
Thioridazine	25, 50, 100 mg every 6 h/21 days (= max. 400 mg/day)	Initially, 50 × 3 mg/day, standard therapy: 200–800/day
Sertraline	Max. 250 mg/day	50–200 mg/day
Tranylcypromine	10–60 mg/day	30 mg/day, max. 60 mg/day
Metformin	Not reported	Max. 2250 mg/day
Pioglitazone	45 mg/day	15–30 mg/day
Pravastatin	40–1680 mg/day	40–80 mg/day
Lovastatin	Escalation 0.5–24 mg/kg/day (~35–1680 mg/day)	Initially, 20 mg/day; maintenance: 10–80 mg/day
Atorvastatin	80 mg/day 1–4 weeks	Max. 80 mg/day
